# Cost-effectiveness of opportunistic QCT-based osteoporosis screening for the prediction of incident vertebral fractures

**DOI:** 10.3389/fendo.2023.1222041

**Published:** 2023-07-31

**Authors:** Sebastian Rühling, Julian Schwarting, Matthias F. Froelich, Maximilian T. Löffler, Jannis Bodden, Moritz R. Hernandez Petzsche, Thomas Baum, Maria Wostrack, A. Kaywan Aftahy, Vanadin Seifert-Klauss, Nico Sollmann, Claus Zimmer, Jan S. Kirschke, Fabian Tollens

**Affiliations:** ^1^ Department of Diagnostic and Interventional Neuroradiology, School of Medicine, Klinikum rechts der Isar, Technical University of Munich, Munich, Germany; ^2^ Department of Radiology and Nuclear Medicine, University Medical Centre Mannheim, Medical Faculty Mannheim-University of Heidelberg, Mannheim, Germany; ^3^ Department of Diagnostic and Interventional Radiology, University Medical Center Freiburg, Freiburg im Breisgau, Germany; ^4^ Department of Neurosurgery, School of Medicine, Klinikum rechts der Isar, Technical University of Munich, Munich, Germany; ^5^ Department of Gynecology, School of Medicine, Klinikum rechts der Isar, Technical University of Munich, Munich, Germany; ^6^ TUM-Neuroimaging Center, Klinikum rechts der Isar, Technical University of Munich, Munich, Germany; ^7^ Department of Diagnostic and Interventional Radiology, University Hospital Ulm, Ulm, Germany

**Keywords:** cost-effectiveness analysis, osteoporosis, opportunistic QCT, fracture prevention, screening

## Abstract

**Objectives:**

Opportunistic quantitative computed tomography (oQCT) derived from non-dedicated routine CT has demonstrated high accuracy in diagnosing osteoporosis and predicting incident vertebral fractures (VFs). We aimed to investigate the cost-effectiveness of oQCT screening compared to dual-energy X-ray absorptiometry (DXA) as the standard of care for osteoporosis screening.

**Methods:**

Three screening strategies (“no osteoporosis screening”, “oQCT screening”, and “DXA screening”) after routine CT were simulated in a state-transition model for hypothetical cohorts of 1,000 patients (women and men aged 65 years) over a follow-up period of 5 years (base case). The primary outcomes were the cumulative costs and the quality-adjusted life years (QALYs) estimated from a U.S. health care perspective for the year 2022. Cost-effectiveness was assessed based on a willingness-to-pay (WTP) threshold of $70,249 per QALY. The secondary outcome was the number of prevented VFs. Deterministic and probabilistic sensitivity analyses were conducted to test the models’ robustness.

**Results:**

Compared to DXA screening, oQCT screening increased QALYs in both sexes (additional 2.40 per 1,000 women and 1.44 per 1,000 men) and resulted in total costs of $3,199,016 and $950,359 vs. $3,262,934 and $933,077 for women and men, respectively. As a secondary outcome, oQCT screening prevented 2.6 and 2.0 additional VFs per 1,000 women and men, respectively. In the probabilistic sensitivity analysis, oQCT screening remained cost-effective in 88.3% (women) and 90.0% (men) of iterations.

**Conclusion:**

oQCT screening is a cost-effective ancillary approach for osteoporosis screening and has the potential to prevent a substantial number of VFs if considered in daily clinical practice.

## 1 Introduction

Effective and widely accessible screening methods that are cost-effective are critical from a clinical and health economics perspective to address osteoporosis as a major public health concern. If left undetected, the silently occurring deterioration in bone microarchitecture often leads to low-impact fractures, most commonly at the spine (1). Osteoporotic fractures (OFs) are associated with high mortality and high re-fracture rates (2–5). Today, in the United States alone, approximately 2 million OFs per year cause more than 800,000 hospitalizations, with subsequent facility-related costs exceeding those of myocardial infarction, breast cancer, or stroke (6). Despite these circumstances, osteoporosis remains under-diagnosed and under-treated (7).

Bone mineral density (BMD) assessment with dual-energy X-ray absorptiometry (DXA) is the recommended standard screening method for postmenopausal women (8). Specifically, DXA has been shown to be cost-effective and can prevent OFs if subsequent treatment is initiated appropriately (9). However, after more than three decades of use, DXA continues to fall short of clinical needs (10, 11). Among others, two fundamental limitations have been identified that limit DXA as an adequate screening method: 1) DXA is a two-dimensional projectional technique that is modestly capable of correctly identifying individuals with osteoporosis (12, 13); 2) DXA is often initiated after the occurrence of an OF when osteoporosis is already fully established. Rates for early DXA screening and the initiation of therapy were initially low and have further declined over the past decades (14–17).

This contrasts with the tremendous increase in computed tomography (CT) examinations and the widespread distribution of CT scanners even in small-scale hospitals (CT scans in the United States: 84.5 million in 2021 vs. 34.9 million in 2000) (18). Each CT scan can potentially provide the quantitative information needed for the accurate detection of osteopenia and osteoporosis via BMD if it is calibrated (19). Over recent years, advances in computational performance, data processing, and the availability of large datasets have promoted the development of fully automated extraction pipelines (10, 11, 20). Here, the extraction of volumetric BMD can be performed in a so-called opportunistic approach using data that have been acquired for clinical indications other than densitometry. This could potentially be used to narrow the diagnostic gap in osteoporosis.

In recent retrospective investigations, opportunistic osteoporosis assessment using automated pipelines has been shown to 1) improve the prediction of prevalent and incident vertebral fractures (VFs) compared to DXA (21, 22) and 2) has demonstrated high diagnostic accuracy for correctly identifying patients with osteopenia and osteoporosis in comparison to dedicated quantitative CT (QCT) (23). Moreover, such an ancillary approach may be seamlessly integrated without disrupting the clinical workflow, does not cause additional radiation exposure, is user-independent, and can be performed prospectively and retrospectively.

Nonetheless, the cost-effectiveness of such an approach regarding VFs has not been evaluated yet. Therefore, the purpose of this study was to investigate whether osteoporosis screening using opportunistic QCT (oQCT) is cost-effective for women and men aged 65 years and older and to identify patient-level cost thresholds.

## 2 Materials and methods

### 2.1 Model overview

This study did not require institutional review board approval. We developed a state-transition model (Markov model) by using established modeling software (TreeAge Pro 2020, TreeAge Software, Williamstown, MA, United States). Our study was conducted from the perspective of the U.S. health care sector, and all costs were measured in United States dollars (USD) (24). Recommendations on discounting costs and outcomes at a discount rate of 3% were followed (25). Based on the WHO-CHOICE recommendations, the willingness-to-pay (WTP) thresholds were set at 1× the gross domestic product (GDP) per capita to indicate high cost-effectiveness and at 3× the GDP per capita to indicate cost-effectiveness (26). Based on 2021 data from the World Bank, the thresholds for the U.S. were $70,249 and $210,746 (27).

### 2.2 Screening population

We simulated hypothetical cohorts of 1,000 women and men aged 65 years and older (base case). We initiated screening at the age recommended by the 2018 U.S. Preventive Services Task Force (USPSTF) for women (i.e., 65 years and older) (8). For cost-effectiveness analyses, an adequate time horizon needs to capture the benefits and harms of the investigated interventions while avoiding unnecessary complexity (28). For the drug therapy options evaluated in this study, reductions in fractures and increases in BMD are best demonstrated over a period of approximately 3–5 years, and the likelihood of side effects or treatment failures was also reported to be the greatest during this period (29, 30). Therefore, the time horizon of our economic model was set to 5 years.

Furthermore, patients did not have any medical condition that would disqualify them for subsequent DXA screening, nor did they have any prevalent OFs, or a history of osteoporosis medication prior to the different screening scenarios. We assumed that all CT scans containing at least the lumbar spine would be evaluable for oQCT screening (e.g., no spinal implants present spanning the whole lumbar spine). Sensitivity and specificity parameters for this specific opportunistic screening method have been documented, in particular for VFs (22, 31). Therefore, we focused on modeling VFs in our Markov analysis.

**Figure 1:**
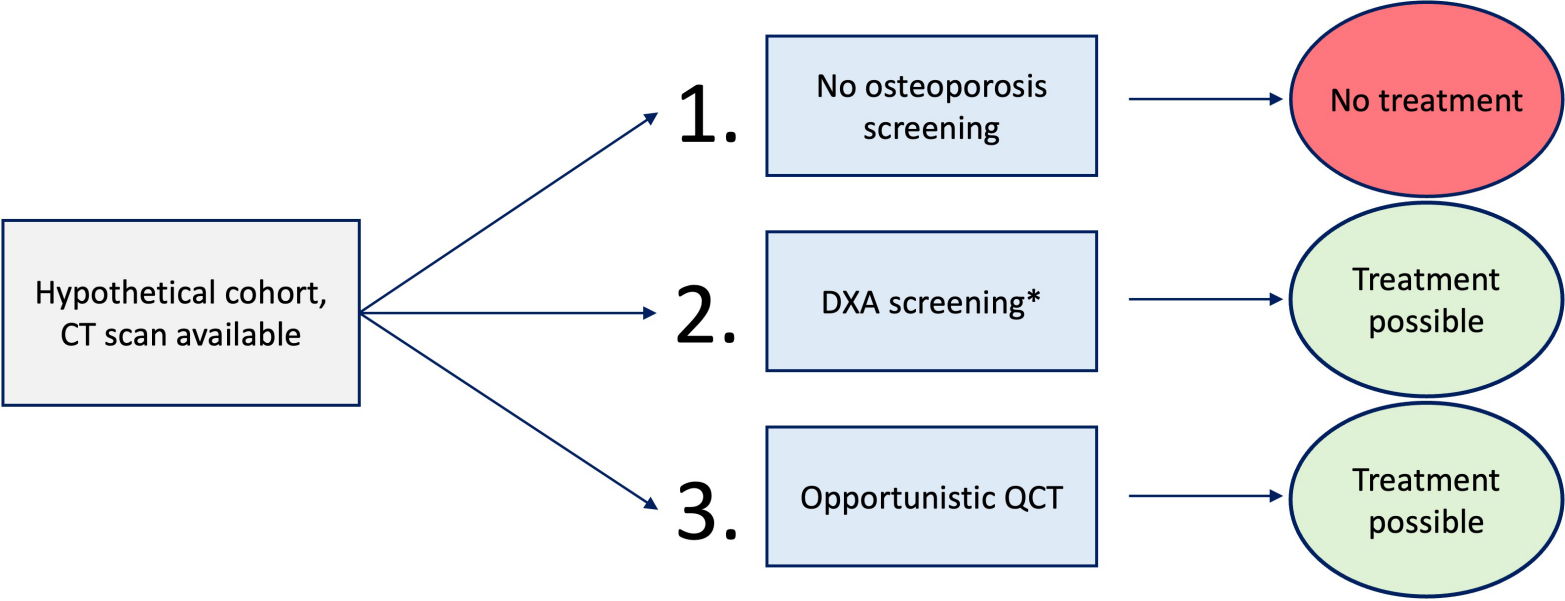
Overview of the three different scenarios. *Annual dual-energy X-ray absorptiometry (DXA) screening rates were applied according to the literature.

The hypothetical cohorts were assigned to three different scenarios (**Figure 1**):

1. No osteoporosis screening. No treatment.
2. No consideration of available CT data. DXA screening, according to reported annual screening rates. Treatment possible.
3. oQCT screening at baseline. Treatment possible.

**Figure 2:**
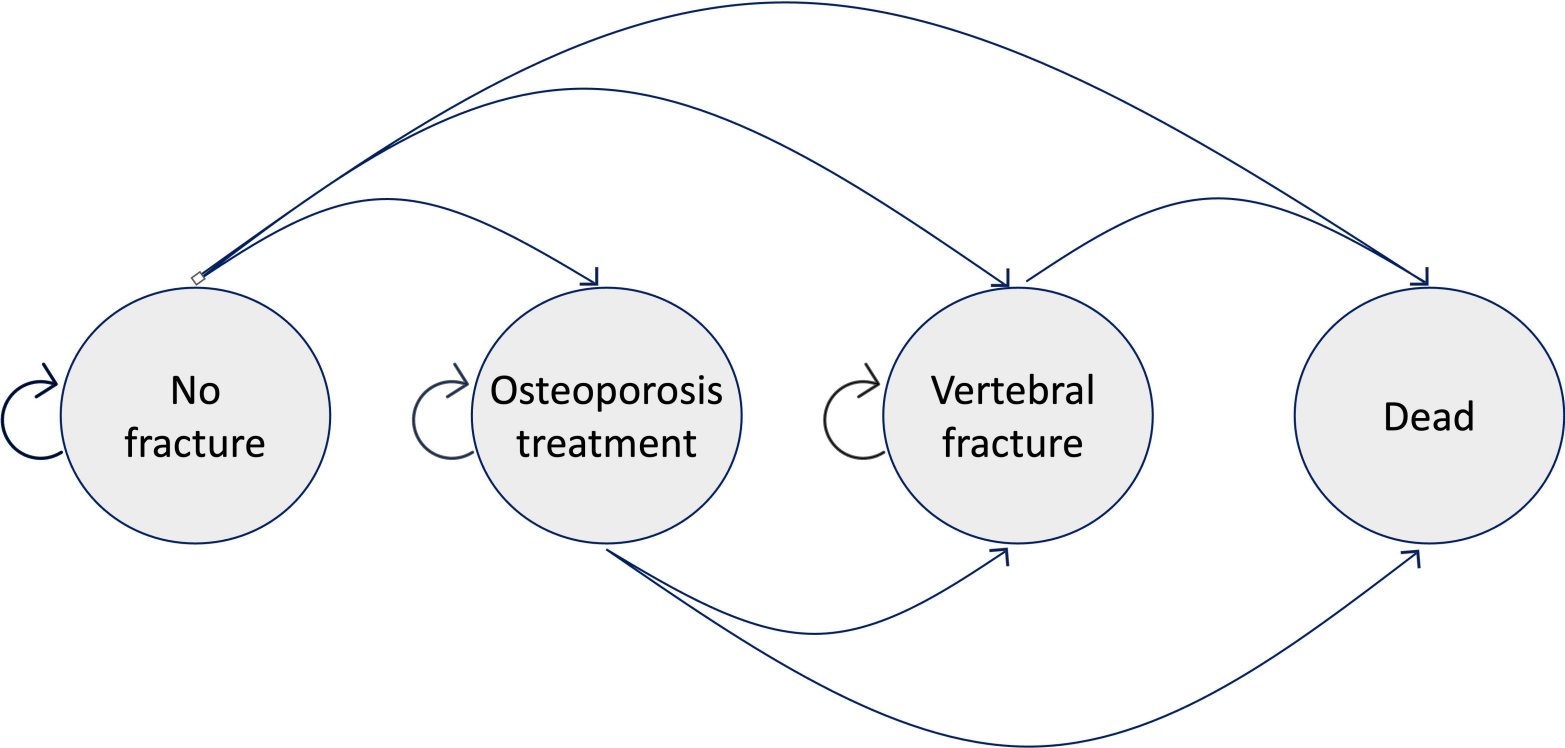
Depiction of the different transition states. All individuals started in the “No fracture” state and transitioned yearly to the other three states or stayed healthy. For simplicity, other sub-states (e.g., re-fracture states) are not shown here.

In each scenario, cohorts transitioned through different health states in annual cycles for the total 5-year period. Our Markov model included the four states “alive without VF”, “alive without VF and on osteoporosis treatment”, “alive with a VF”, and “dead” (absorbing state) (**Figure 2**). The model estimated direct costs, quality-adjusted life years (QALYs), the incremental cost-effectiveness ratio (ICER), and the number of VFs.

### 2.3 Input variables

Input variables for this economic evaluation were collected from recent literature (**Table 1**) closely following international recommendations on the conduct, methodology, and reporting of cost-effectiveness analyses (25, 47). The sources of the data are further explained in detail below.

**Table 1:** Key model parameters for the Markov model.

Parameter	Value	Range	Distr.	Source
Starting age	65 (W) 65 (M)			Curry et al. (8) Asm.
oQCT screening				
Sensitivity Specificity Screening rate (year 1)	0.59 (W/M) 0.81 (W/M) 1.0	0.5–0.7	β β β	Löffler et al. (31) Löffler et al. (31) Asm.
DXA screening				
Sensitivity/specificity Screening rates (years 1–5)	0.565/0.688 (W) 0.215/0.967 (M) 0.095 (W) 0.017 (M)	0.5–0.65 0.15–0.4 0.04–0.15 0.005–0.030	β β β β	Iki et al. (32) Chalhoub et al. (33) Zhang et al. (34) Zhang et al. (34)
Risks				
Incidence of VFs (per 1,000 patients) 65–75 years	17.0 (W) 5.1 (M)	12.0–22.0 3.5–6.5	β β	Van Der Klift et al. (35) Van Der Klift et al. (35)
Probability of dying after a VF (age 60)				
Year 0 Year 1 Year 2 Year 3 Year 4 Year 5	0.129 (W)/0.134 (M) 0.103 (W)/0.107 (M) 0.083 (W)/0.085 (M) 0.066 (W)/0.068 (M) 0.053 (W)/0.054 (M) 0.043 (W)/0.043 (M)		β β β β β β	Johnell et al. (36) Johnell et al. (36) Johnell et al. (36) Johnell et al. (36) Johnell et al. (36) Johnell et al. (36)
Probability of dying from other causes				
65–66 66–67 67–68 68–69 69–70	0.009638 (W)/0.016078 (M) 0.010386 (W)/0.017216 (M) 0.011235 (W)/0.018401 (M) 0.012237 (W)/0.019666 (M) 0.013393 (W)/0.021099 (M)		β β β β β	Arias et al. (37) Arias et al. (37) Arias et al. (37) Arias et al. (37) Arias et al. (37)
RR of VF with bisphosphonate treatment	0.55 (W) 0.44 (M)		β β	Byun and Black et al. (38, 39) Zeng et al. (40)
RR of re-fracture after sustaining a VF				
Year 1 Years 2–5	5.0 (W/M) 2.5 (W/M)		β β	Lindsay et al. (5) and Asm. Van Geel et al. (2) and Asm.
Costs				
oQCT screening DXA screening	$82,61 $111,19	Variable ± 30%	γ γ	Medicare Services (41) Medicare Services (41)
Costs of VF (Medicare insurance)	(inflated to 2022 $)			
Year 1 Year 2 Year 3 Year 4 Year 5 Yearly treatment costs Annual discount rate	$24,012 $5,958 $4,234 $3,033 $2,245 $100 3%		γ γ γ γ γ γ	Tran et al. (42) Tran et al. (42) Tran et al. (42) Tran et al. (42) Tran et al. (42) Federal Supply Schedule (43) Sanders et al. (25)
Treatment				
Treatment duration Adherence to treatment	2 years 0.5	0.3–0.7	β	Kothawala et al. (44)
Utility				
Utility weights	0.84 (W) 0.87 (M)		β β	Fryback et al. (45) Fryback et al. (45)
Disutility multiplier				
First year Subsequent years	0.860 0.965		β β	Hiligsmann et al. (46) Hiligsmann et al. (46)

Distr., distribution; VF, vertebral fracture; RR, relative risk; Asm., assumption; oQCT, opportunistic quantitative computed tomography; DXA, dual-energy X-ray absorptiometry.

### 2.4 Diagnostic accuracy parameters

Several studies have shown that CT-based BMD may outperform DXA in the prediction of prevalent and incident VFs (31, 33, 48, 49). In a recent study, oQCT using asynchronous calibration demonstrated high diagnostic accuracy comparable to dedicated QCT (23). For the prediction of incident VFs for both sexes, sensitivity and specificity parameters were adopted from a previously published single-center study validating the same technique for VF prediction in direct comparison to DXA in a comparable cohort (31). Here, patients tested positive if the BMD was below the diagnostic cutoff for osteoporosis (<80 mg/cm^3^) as defined by the American College of Radiology (19). Other multicenter prospective cohort studies have reported similar area under the curve (AUC) values for the predictability of VFs with dedicated QCT (33, 49). For DXA, the sensitivity and specificity parameters were based on 1) the Japanese Population-Based Osteoporosis Study (JPOS) for women and 2) the multicenter Osteoporotic Fractures in Men Study (MrOS) (32, 33).

### 2.5 Probability of fractures and therapy effects

The sex- and age-adjusted incidence rates for VFs were taken from the prospective population-based Rotterdam study (35). We assumed the incidence rates as the background risk of the population, also to account for the distribution of modifiable risk factors (e.g., glucocorticoid use or cigarette smoking). To date, a variety of pharmacologic treatment options exist for osteoporosis and osteopenia (50). In this study, we modeled the benefits of the established and widely investigated bisphosphonates (i.e., alendronate). We assumed the adherence to the treatment to be 50% over the 2 years of treatment duration, according to respective meta-analyses (44, 51). In the subgroups of high-risk patients identified by DXA (i.e., lumbar T-score at or below −2.5) or oQCT (i.e., BMD < 80 mg/cm^3^) who were consequently treated with bisphosphonates, the risk of subsequent VFs was reduced according to published meta-analyses (38–40).

### 2.6 Cost estimates

Taking a U.S. health care perspective, the model simulated direct medical expenditures. We did not consider societal, indirect, and individual patient costs. Costs were inflated to 2022 USD using the consumer price index by the U.S. Bureau of Labor Statistics (52). Costs of DXA were set to $111,19 and to $82,61 for oQCT screening based on Medicare Procedure Codes 77080 (DXA, bone density study, one or more sites, and axial skeleton) and 77078 (CT, bone mineral density study, one or more sites, and axial skeleton) (41). In the case of a VF, adjusted differences in total all-cause direct health care costs compared to matched non-fracture controls were applied (42). The annual costs for a patient decreased dynamically in years 1–5 after the fracture event (**Table 1**). Treatment costs for oral administration of generic alendronate were estimated at $100 per year (43).

### 2.7 Transition probabilities

The average age-specific risk of death was extracted from U.S. life tables (37). A dynamically decreasing risk of mortality in the years following a VF was extracted from the literature (36). Individuals with a VF could also enter the re-fracture state, and dynamic risk variables for increased risk of additional fractures were included (2, 5).

### 2.8 Outcome estimates

Estimates of the quality of life of women and men aged 65 to 74 years and disutility multipliers for VFs stratified for the first year after VF and subsequent years were derived from the literature (45, 46). Patients without VFs and patients receiving osteoporosis treatment were assumed to have an unimpaired quality of life.

### 2.9 Sensitivity analyses

**Figure 3:**
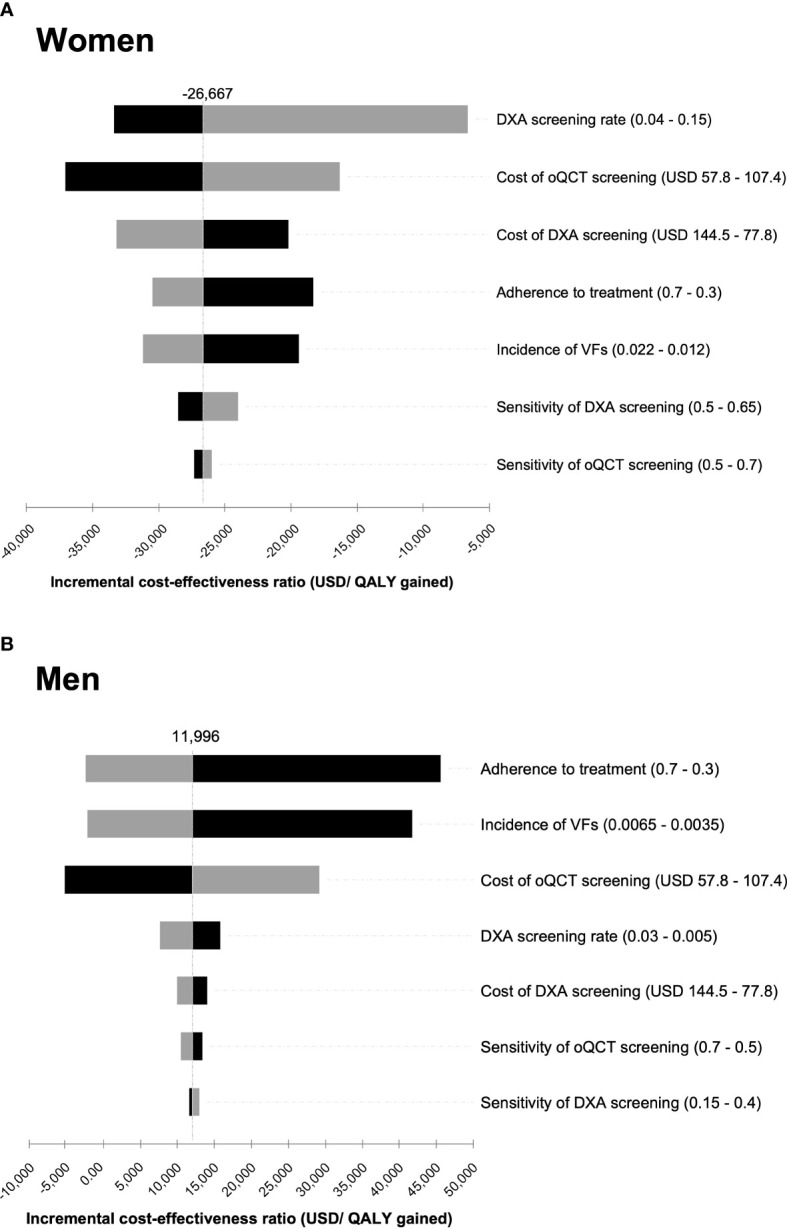
Tornado diagram of the deterministic sensitivity analysis for women **(A)** and men **(B)**. Costs and sensitivity of the diagnostic procedures, dual-energy X-ray absorptiometry (DXA) screening rate, incidence, and adherence to treatment were varied within a reasonable range to illustrate their impact on the incremental cost-effectiveness ratio (ICER) of opportunistic quantitative computed tomography (oQCT) screening compared to DXA screening. The range in which each parameter was varied is indicated in brackets behind each parameter name. The different bars represent the changes in the ICER for each input parameter, starting from the base case. Top bars represent parameters that contribute most to the variability of the ICER. The bar colors represent high (gray) and low (black) values for each parameter.

**Figure 4:**
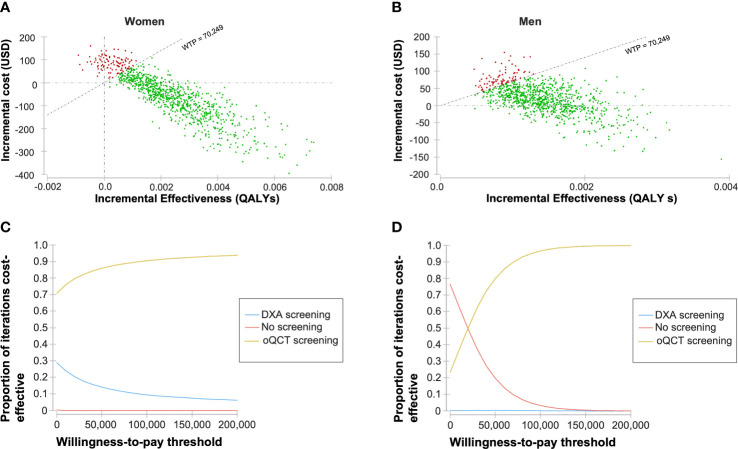
Probabilistic sensitivity analysis based on 30,000 Monte Carlo simulations. Incremental costs and effectiveness comparing opportunistic quantitative computed tomography (oQCT) to dual-energy X-ray absorptiometry (DXA) screening in women **(A)** and men **(B)**. A willingness-to-pay (WTP) threshold of 70,249 USD per quality-adjusted life year (QALY) gained was assumed. Resulting acceptability curves for women **(C)** and men **(D)**.

Deterministic sensitivity analyses were conducted to estimate the impact of varying input variables on the model outcomes. Input variables were varied within predefined ranges (**Table 1**). The influence of the variation of the input variables on the ICER was visualized in the form of a tornado diagram (**Figure 3**). Probabilistic sensitivity analyses and Monte Carlo simulations with 30,000 iterations were conducted to test the model’s stability (**Figure 4**).

## 3 Results

### 3.1 Cost-effectiveness analysis

The base case analysis of 1,000 women resulted in cumulative costs of $3,199,016 and $3,262,934 with 4,516 and 4,513 QALYs over the 5-year period for oQCT screening and for DXA screening, respectively (**Table 2**). Comparing DXA screening to no screening, and oQCT screening to DXA screening, 4.4 VFs and 2.6 VFs could be averted in women, respectively.

**Table 2:** The cumulative discounted costs in USD ($), outcomes (QALYs), and prevented VFs calculated for a cohort of 1,000 women and men for a time frame of 5 years.

Strategy	Cumulative discounted costs ($)	Incremental costs ($)	Cumulative discounted effectiveness (QALYs)	Incremental effectiveness (QALYs)	Vertebral fractures (VFs)	Incremental cost-effectiveness ratio ($/QALY)
No osteoporosis screening						
Women	3,361,620	162,604	4,511	−4.52	90.7	–
Men	924,747	–	4,636	–	25.6	–
DXA screening						
Women	3,262,934	63,918	4,513	−2.40	86.3	–
Men	933,077	8,331	4,636	0.03	25.5	290,542
oQCT screening						
Women	3,199,016	–	4,516	–	83.7	–
Men	950,359	25,612	4,638	1.47	23.5	17,432

DXA, dual-energy X-ray absorptiometry; QALYs, quality-adjusted life years; VF, vertebral fracture; oQCT, opportunistic quantitative computed tomography.

In men, simulation of a 5-year period resulted in cumulative costs of $950,359 and $933,077 with 4,638 QALYs and 4,636 QALYs for oQCT screening and DXA screening, respectively. Comparing oQCT to DXA screening, an ICER of $11,996 per QALY was obtained, and two VFs were averted.

### 3.2 Sensitivity analysis

**Figure 5:**
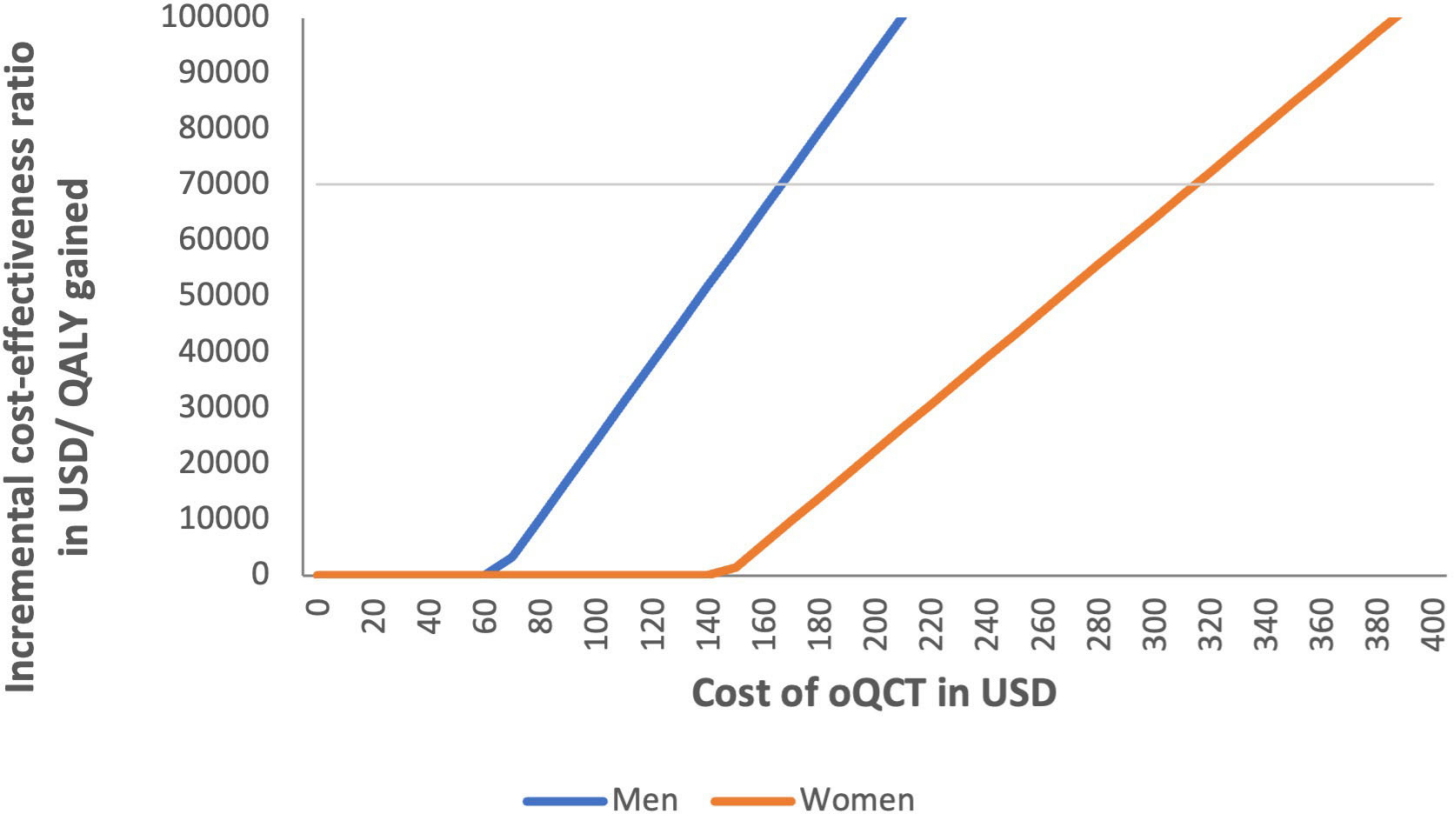
Impact of varying costs of opportunistic quantitative computed tomography (oQCT) screening on incremental cost-effectiveness ratio (ICER) of oQCT screening compared to dual-energy X-ray absorptiometry (DXA) screening. oQCT was cost-effective for costs up to $315 and $167 in women and men, respectively. oQCT was cost-saving for costs below $140 and $60 for women and men, respectively.

ICERs were examined for varying costs for oQCT screening compared to DXA screening. When the costs of oQCT screening were smaller than $315 and $167, oQCT screening was cost-effective in women and men, respectively (**Figure 5**). When the cost was reduced below $140 and $60, oQCT screening of women and men was cost-saving.

In the deterministic sensitivity analysis, variations in DXA screening rate, cost of screening, and adherence to treatment significantly affected the resulting ICERs in the screening of women, whereas the sensitivity of DXA and oQCT screening had only a minor influence on cost-effectiveness (**Figure 3**). For men, adherence to treatment, incidence of VFs, cost of oQCT screening, and DXA screening rate were most influential, whereas sensitivity of DXA and oQCT screening had only a minor influence on cost-effectiveness. Varying these input variables within reasonable ranges resulted in ICERs below $50,000 per QALY gained for men.

In the vast majority of Monte Carlo iterations, oQCT screening was cost-effective (**Figure 4**). At a WTP threshold of $70,249 per QALY gained, 88.3% and 90.0% of the iterations were cost-effective for women and men, respectively.

## 4 Discussion

This study investigated the cost-effectiveness of oQCT-based osteoporosis screening in clinical routine compared to standard-of-care DXA screening. While other studies have already addressed this for various other OF types, we focused on VFs and estimated cost thresholds for opportunistic screening algorithms (53). Here, we were able to confirm the cost-effectiveness of oQCT as a screening tool for asymptomatic male patients at designated risk for VFs. In women, oQCT was even cost-saving (i.e., the total costs would be lower than without screening) and the most effective strategy. Furthermore, compared to standard care, applying oQCT prevented a substantial number of VFs in both women and men.

Several studies have performed cost-effectiveness analyses to evaluate DXA- and CT-based screening strategies followed by bisphosphonate therapy (9, 53–56). Nayak et al. found that DXA screening strategies with different (re-)screening intervals and target populations (women aged 55 years and older and men aged 50 years and older) were cost-effective compared to no screening at a lifetime horizon (9, 54). Considering a 5-year time window, reported screening rates, and outcomes related to VFs only, we were able to show the cost-effectiveness of standard-of-care DXA screening in women but not in men. Other studies have tested biomechanical computed tomography (BCT) against DXA screening, no screening, or in combination (55, 56). While Agten et al. reported an ICER of $2,000/QALY for the combination of DXA and dedicated BCT in postmenopausal women for all fracture types at a lifetime horizon, Pisu et al. reported cost-saving scenarios for BCT on existing CT scans for both sexes in relation to hip fractures at a 5-year horizon (55, 56). While in our model oQCT screening in men was not cost-saving, the reported ICER of $17,432 per QALY was below accepted WTP thresholds.

In this study, we applied annual incidence rates from the large prospective Rotterdam study to our hypothetical cohorts (35). A recent study examining the prevalence of VFs in a patient population more comparable to our scenario (i.e., elderly patients in clinical routine receiving CT scans) found a prevalence of VFs of up to 30% (57). Thus, the typical patient population, as well as our hypothetical cohorts, can be considered a risk collective to some extent, and we assume that our incidence rates were conservative. Our finding that the cost-effectiveness of oQCT was sensitive to VF incidence rates, particularly in men but also in women, further indicates the importance of screening in high-risk populations.

Our model only simulated the potential benefits of prevented VFs. We did not consider the automated detection of prevalent VFs, for which pharmacological treatment would also be recommended to patients. In this context, it should be noted that QCT at the spine also provides information about hip fracture risk (19, 33). These potential benefits, as well as the potential prevention of all other OFs in addition to VFs, were not addressed in our simulation model and could further improve the cost-effectiveness of opportunistic screening.

Deterministic sensitivity analyses revealed that the cost and sensitivity of DXA screening had only a minor influence on the resulting cost-effectiveness in men. The generally low annual DXA screening rate of 1.7% in men likely contributes to this finding and may explain why variations in DXA cost or sensitivity have a minor influence on resulting overall costs and effects (34). Importantly, the costs of oQCT screening, adherence to treatment, and incidence of VFs can considerably impact cost-effectiveness in both sexes. As a consequence, the economic value of oQCT screening could be improved by increasing the adherence to bisphosphonate treatment, which was assumed at 50% in the present analysis, and by pre-selecting patients for oQCT screening, e.g., based on pre-existing clinically available data, to increase the pre-test probability of osteoporosis. In this regard, fracture liaison services, which have also proven to be cost-effective, are a promising option to increase treatment adherence (58, 59).

The costs of oQCT analyses are currently not reflected in the Medicare program. We therefore used the cost of a dedicated CT bone density scan ($82,61) in the base case analysis. When the costs of oQCT did not exceed $315 in women and $167 in men, oQCT screening remained cost-effective. Compared to the cost of a dedicated CT bone density scan, it can be assumed that the costs of oQCT screening software could very likely fall below the cost thresholds identified. We found that breakeven costs for the oQCT scenario (i.e., equal overall costs for the oQCT scenario compared to DXA screening) were $147 per evaluated scan by oQCT for women and $65 for men.

The USPSTF has not yet endorsed recommendations for or against osteoporosis screening in men (8). For women, the USPSTF lists two main action items for the prevention of OFs: 1) DXA bone measurements in women aged 65 years and older and 2) the application of clinical risk tools for younger women, followed by a subsequent DXA testing when appropriate (8). Clinical risk assessment scores (e.g., FRAX) are non-invasive, powerful, and cost-effective tools to easily identify patients at risk, as recommended by the International Osteoporosis Foundation (IOF) (60). In addition, FRAX can also be applied if there is no BMD available. Nevertheless, in a recent study of women, 94.1% of patients at increased risk of fragility fractures were untreated (7). In comparison, this treatment gap was 63% lower in a group of patients with a definite diagnosis of osteoporosis (7). For a definite diagnosis, bone mass is currently the most crucial determinant in addition to the occurrence of atraumatic fractures, also to monitor treated patients. However, the need for BMD assessment contrasts with the under-resourcing (i.e., equipment and utilization) not only in the United States but also in many European countries (61). While it remains unclear whether national efforts can re-increase DXA screening rates, thousands of CT scans are performed daily in routine clinical practice worldwide. In this context, the decentralized nature of oQCT could add true value by providing physicians with immediate BMD estimates for diagnosis and fracture prediction, thus narrowing the diagnostic gap that precedes the treatment gap.

For the successful implementation of screening methods, attainable technical requirements and their availability across different health care systems play a vital role. Whereas conventional QCT requires simultaneous scanning of a dedicated calibration phantom, oQCT uses conversion factors based on asynchronous phantom measurements that allow for prospective and retrospective measurements (10, 11). When other well-known confounding factors (e.g., tube voltage and administration of intravenous contrast agents) are considered, oQCT has demonstrated high accuracy compared with dedicated QCT in a variety of clinical settings (23, 62). Although the oQCT technique is still evolving, a fully automated pipeline for opportunistic BMD measurements has recently been shown to outperform DXA in the prediction of prevalent and incident VFs (21, 22). While further prospective validation studies are clearly needed, such frameworks could be applied to large patient populations given the minimal user interaction required.

There are several limitations to our study. First, the Markov model used for our analyses only allows estimates of sequential disease states and associated costs and benefits. While most data (e.g., on fracture incidence or recurrence risk) were retrieved separately for women and men, some data (e.g., on dynamic fracture costs) were based on studies conducted only in women (42). Therefore, the model can never fully reflect clinical reality. Second, whereas the DXA parameters for sensitivity and specificity for predicting VFs were based on large prospective cohorts, the parameters for oQCT screening were based on a rather small sample size for both sexes (31–33). Although a larger sample size would be preferable, the reported AUC is within the range of other studies investigating the predictability of VFs with dedicated QCT (33, 49). For our study, we used the parameters reported by Löffler et al. with an AUC of 0.76 for oQCT screening in both sexes (31). For dedicated QCT, Wang et al. reported an AUC of 0.82 and Chalhoub et al. had an AUC of 0.79, both in men (33, 49). Nevertheless, extension studies with larger sample sizes are currently being conducted to rule out the possibility that the results of oQCT screening in the current simulation study may be exaggerated. To rule out a potential population bias, the parameters used for DXA in women were compared with other studies in White populations (which unfortunately reported only AUC values) (32, 63, 64). As an example, Hans et al. described AUC values for DXA prediction of VFs in a large Canadian female population (AUC of 0.69), comparable to the Japanese population-based study used for the Markov model (AUC of 0.673). Third, potential harm from drug treatment (e.g., bisphosphonates) was not considered, as the risk of treatment-related adverse events is considered low after decades of use (8). Assuming that oQCT is performed on existing CT data, we also did not consider potential radiation risks, and thus, our study cannot be extrapolated to dedicated QCT. Fourth, our input parameters for oQCT were based on calibrated scans (i.e., asynchronous calibration). Therefore, our results are not applicable to screening techniques that use only uncalibrated CT attenuation values.

## 5 Conclusion

We confirmed CT-based opportunistic osteoporosis screening in our model as a cost-effective ancillary option also for VF prevention in asymptomatic patients, determined cost thresholds for a reasonable and cost-effective application of oQCT screening, and identified major determinants that could be optimized to further improve the cost-effectiveness of oQCT screening.

## Data availability statement

The raw data supporting the conclusions of this article will be made available by the authors, without undue reservation.

## Ethics statement

Ethical review and approval were not required because the data was retrieved by previous publications which had a respective waiver or approval of the institutional review board.

## Author contributions

SR, FT, and JK conceptualized and designed the study. JS and MF contributed to the concept and design of the study. SR and JS organized the database. JB, ML, MH, MW, KA, and TB contributed to the generation and organization of the database. FT developed the Markov model. SR and JK contributed to the development of the Markov Model. SR, FT, JK and JS performed the formal and statistical analysis. SR and FT wrote the manuscript. ML, JS, MF, JB, MH, TB, MW, KA, VS-K, CZ, NS, and JK critically revised the manuscript. CZ, JK, VS-K, and MF provided scientific supervision and resources. JK and CZ provided funding. All authors contributed to the article and approved the submitted version.
